# The Sanitary Commission

**Published:** 1864-09

**Authors:** 


### THE SANITARY COMMISSION.

The present war, terrible in its magnitude and duration, has had benevolent accompaniments never before known in the history of the world. The various organizations for the relief of soldiers, sick and wounded, in the camp, the field and the hospital, which are planted squarely on the generosity and patriotism of the people, brighten the gloom which our three years of conflict has hung around our country. Foremost among these is the Sanitary Commission, which has become a gigantic humanitarian organization, for the relief of the sick and wounded of our army. Only those intimate with its immense operations can utter its eulogy, or realize one-half its beneficient work.

The chief object contemplated by the Sanitary Commission, when it was created by the Government, was the prevention of disease. Modern sanitary science was hardly recognized in the ancient regulations of the medical bureau, and consequently, during the summer of 1861 our armies were in serious danger of destruction from epidemic disease. The first business of the Commission, therefore, was to awaken general attention to the sanitary interests of the army, and to do what it could to improve the sanitary condition of camps, hospitals, and men. It brought to bear upon Government the influence of the medical profession throughout the country, effected the extension and invigoration of the medical bureau, and secured the express recognition of the prevention of disease, no less than its cure, as among the functions of the medical staff. Government now employs its own sanitary inspectors, and does a certain portion of the preventive work which the Commission performed during the first year of its existence. But the Commission find it necessary to keep up an inspectorial corps likewise.

The visits of the inspector usually disclose something that can be done to improve or promote the health of the command. He finds that quinine is necessary to prevent malarious disease, or vegetables to prevent scurvy, or that stimulants, bedding, disinfecting agents, or hospital diet, are wanted. In consequence of his reports, the Commission have dug wells, to improve the water-supply of camps, improved the ventilation of hospitals, built temporary hospitals and quarters to replace unwholesome and dangerous buildings, furnished and fitted up hospital transports, and converted ordinary railroad cars into railroad ambulances, with cooking apparatus and store-rooms, and litters hung on springs, in which thousands of men with fractured limbs have traveled thousands of miles without injury.

It has furnished material for the vaccination of thousands of men at a time, when the medical bureau was not able to supply immediately what was needed. It has circulated throughout the army, and especially among the medical staff, many hundred thousand of its medical documents. These have been prepared by some of the most eminent surgeons and physicians of the country, and embody, in a condensed form, the latest results of science. They have been of great use to our army surgeons, who often encounter cases for which their previous practice has not prepared them, and who have neither medical libraries nor opportunities for consultation.

It is, however, through the Relief Department of the Sanitary Commission that it is best known to the people. In this work it aims, not to supplant, but to supplement the Medical Department of the Government, to meet exigencies for which the Government has not provided, and to furnish such supplies as the Government does not and can not. When the Medical Purveyor has the articles needed in the hospital and camp, the army draws them from him; when he is out, or when from some emergency a larger supply is needed than is provided by Government, or when because of some technical informalities in the manner of making out requisitions the Purveyor is obliged to refuse them, and thus endanger life by the delay, the army calls upon the Commission, which never fails to respond to the call.

The relief agents of the Commission keep up with the army as it moves forward. Where the army encamps in the morning, the Commission has pitched its tents long before night. It reaches a new base as soon as there are soldiers to protect it, and is at work establishing hospitals and providing necessary stores before the ponderous machinery of the Government has moved; its red flags are seen everywhere at the front, blending with the stars and stripes, where it is establishing its feeding stations and depots of supplies. Prominent and experienced agents accompany each division of the army, with organized corps of assistants, wagons and supplies.

It is at great battles that the agents of the Commission are eminently useful. The battle-service of the Commission requires large funds and supplies. At Murfreesboro, Antietam, Gettysburg, Chattanooga, Vicksburg, and Port Hudson, sudden and vast demands were made. Fifty thousand dollars would not cover the outlay of the Commission the first two weeks after one of our great battles. At Gettysburg it was $75,-000. The average cost is $3.20 to a man; at Gettysburg it wa $10 per man. The outlay of the Sanitary Commission during the months of May and June, for the battle necessities of the present campaign, was over $500,000, exclusive of the supplies directed to it, and which it distributed.

This service requires comprehensive forethought, prompt and energetic action, and unwearied labor in infinite detail. Of some articles the requirements are enormous. Condensed milk, and extract of beef by the ton; wines and spirits by the barrel; crackers and farinaceous food by the ton; tea, coffee and sugar by the chest and hogshead; cargoes of ice; potatoes, onions, pickled cabbage, sourkrout, lemons and oranges, by car loads; shirts, drawers, sheets, crutches, bed-rests and mattresses, by tens of thousands! And this material has to be transported by wagon trains from one base to another; forage for horses has to be provided; drivers have to be paid, steamers chartered, and coal consumed; and yet so economically is the whole work of distribution managed that its cost is less than three per cent.

In addition to this, the Commission supports twenty-five Soldiers' Homes or Lodges, scattered over the whole field of war from New Orleans to Washington; and twenty-three hundred soldiers a-day are taken care of in these homes. Multiply 2,300 by 365, and you have over 800,000 men thus relieved annually. Three other agencies to secure the soldiers' rights are maintained by the Commission: 1. Claim agency to secure his bounty; 2. A pension agency; 3. A back-pay agency—all of them giving their services to the soldier free of charge. Often $20,000 back-pay is secured in one day. A Hospital Directory is also sustained, by which the whereabouts of the sick men is determined, when they are lost to their friends. It costs $20,000 a-year to maintain it, but is worth a million, if the relief afforded to human anxiety can be estimated in money.

To carry on this vast human machinery, the Commission employs 200 agents at an average of about $2 per day—less than an ordinary mechanic's wages—or a total of $12,000 per month, who operating from Texas to the Potomac, or from Charleston to Kansas, and the results are such as to justify the nation's pride in this grand and beneficent, yet truly American institution.—*People's Journal of Health.*

